# Trending of small bowel adenocarcinoma research from 2000 to 2022: A bibliometric analysis

**DOI:** 10.1097/MD.0000000000037795

**Published:** 2024-04-12

**Authors:** Li Li, Shao Zhang, Guang Fu

**Affiliations:** a The First Affiliated Hospital, Department of Gastrointestinal Surgery, Hengyang Medical School, University of South China, Hengyang, China.

**Keywords:** bibliometric analysis, cancer, citespace, small bowel adenocarcinoma, VOSviewer

## Abstract

Small bowel adenocarcinoma (SBA) is a rare tumor entity with a relatively poor prognosis. Diagnosis and management of SBA are still challenging despite recent advancement of diagnostic methods and publication of guidelines. This study aimed to analyze and visualize the trending of SBA research in the past 22 years in the 21st century through bibliometric analysis. Our study collected 1270 publication records of SBA from 2000 Jan 1st to 2022 December 31 from Web of Science and used VOSviewer and CiteSpace to analyze countries, institutions, journals, authors, references and keywords to present the latest trends in SBA research. The USA was the most productive country in terms of the total number of publications (n = 418). The Mayo Clinic (n = 22) and University of Texas MD Cancer Center (n = 22) were the institutions with top publications. The “*World Journal Of Gastroenterology*” (n = 30) had the largest publications. Overman Michael J (n = 17) was the most active and prolific author. The “small bowel adenocarcinoma” was the most frequent keyword. Our bibliometric analysis provides a comprehensive overview of the trends and gaps in the research of SBA. Despite the challenges faced, researchers from USA, Japan and China have made significant contributions to the field of SBA research, and further research is necessary to develop evidence-based guidelines, and advance the understanding and management of SBA.

## 1. Introduction

Small bowel adenocarcinoma (SBA) is a rare but aggressive malignancy and only accounts for <5% of all gastrointestinal cancers and is associated with a poor prognosis due to its late diagnosis and aggressive nature.^[1]^ SBA occurs more commonly in older adults, and was most commonly found in duodenum (50%), followed by jejunum (30%) and ileum (10%).^[2–4]^

Diagnosis of SBA is challenging due to the nonspecific symptoms that patients often present with. These may include abdominal pain, nausea, vomiting, diarrhea, and weight loss.^[3]^ Diagnostic tools for SBA include imaging studies such as computed tomography (CT) scans, magnetic resonance imaging, and endoscopic ultrasound. Tissue biopsies obtained through endoscopy or surgery may also be used to confirm a diagnosis of SBA. However, none of those diagnostic methods were sensitive enough to detect SBA at early stage.^[5,6]^ As a result, SBA is frequently diagnosed at an advanced stage, limiting treatment options and reducing the likelihood of a successful outcome.^[7]^

The treatment of SBA typically involves surgical resection of the tumor, followed by adjuvant chemotherapy and/or radiation therapy. However, due to the aggressive nature of SBA and its propensity for metastasis, the effectiveness of these treatments is limited. The prognosis for SBA is poor, with a 5-year survival rate of approximately 30%.^[1,7]^ Prognostic factors include tumor size, location, and stage at diagnosis.^[1,7]^ Early detection and aggressive treatment are critical to improving outcomes for patients with SBA.

Due to its rarity, SBA is poorly understood compared to other cancers, with limited research and clinical guidelines available.^[8]^ This has led to a lack of consensus on the optimal management of SBA, including the role of surgery, chemotherapy, and radiation therapy.^[8]^ There is also a need for a better understanding of the molecular and genetic characteristics of SBA, which could potentially lead to the development of more targeted therapies.^[9–12]^

Bibliometric analysis is a quantitative method of analyzing scholarly literature and publications. It can provide insight into the landscape of research on SBA by identifying trends in publication output, research topics, and collaborations between researchers and institutions.^[13]^ The aim of this study was to perform a bibliometric analysis of SBA literature from 2000 to 2022 to explore trends in research and identify potential knowledge gaps.

## 2. Methods

We conducted a database search of the Science Citation Index Expanded of Web of Science Core Collection for articles between January 1, 2000, and December 31, 2022, using “small bowel adenocarcinoma” as Topic. We initially retrieved 1606 publications and then refined publications by setting the Language as “English” and Publication type as “Article” and “Review.” Eventually, only 1270 publications were included in the downstream analysis. Full records and cited references for the publications retrieved from the database were exported to.txt format or.CSV file. Next, those data were imported to VOSviewer version 1.6.19, Citespace version 6.2 R3 for visualization analysis. This study was approved by the medical ethics committee of The First Affiliated Hospital, University of South China.

## 3. Results

**Figure 1.**
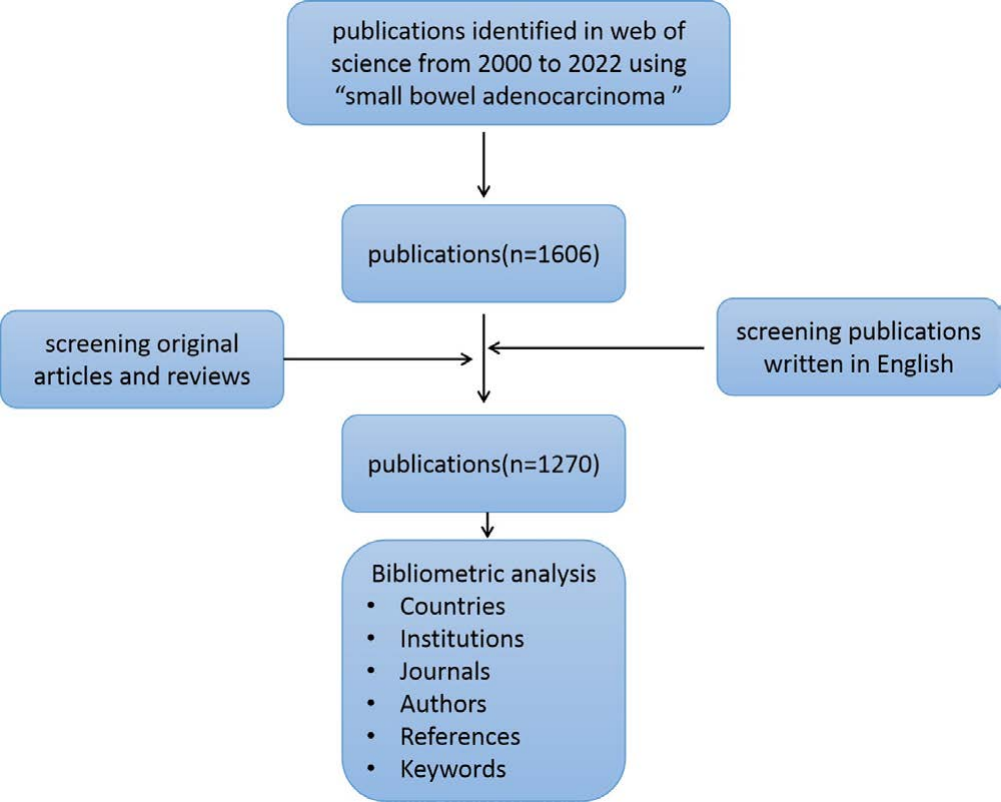
Illustration of search, selection and analysis of publications.

**Figure 2.**
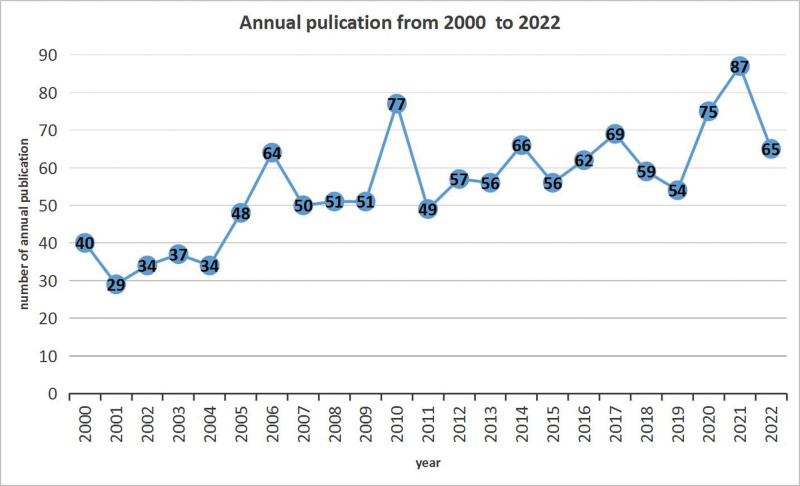
Trend of annual publication of small bowel adenocarcinoma.

Our search identified a total of 1270 articles on SBA from 2000 to 2022 (Fig. 1), with an average annual publication rate of 57.72 articles. The number of publications has steadily but slowly increased throughout the study period. The highest number of articles (n = 94) was published in 2021 and no publication burst was observed (Fig. 2).

**Figure 3.**
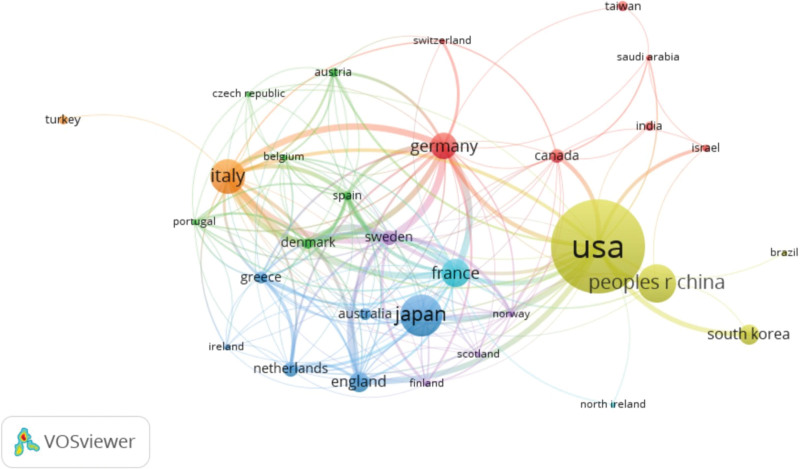
Network map of co-occurring countries in research of small bowel adenocarcinoma.

We then analyzed the geographic distribution of those publications. We found the USA was the top country in terms of the total number of publications (n = 418), followed by Japan (n = 145), China (n = 117), Italy (n = 103), GERMANY (n = 77), and FRANCE (n = 75) (Table 1). Noticeably, USA not only had the largest number of publications, but also had wide collaborations with other countries (Fig. 3). In contrast, although Japan, China and Italy had large publications, they had limited international collaborations (Fig. 3). Those results indicated that the USA played a leading role in research of SBA.

**Table 1:** Top 10 countries with the most publications in research of small bowel adenocarcinoma.

Rank	Country	Publications	Citations	Citations/Publications
1	USA	315	10805	34.30
2	Japan	112	1693	15.12
3	China	102	963	9.44
4	Italy	89	1733	19.47
5	France	68	2429	35.72
6	Germany	62	1591	25.66
7	South Korea	46	633	13.76
8	England	45	1177	26.16
9	Sweden	29	952	32.83
10	Netherlands	29	605	20.86

Countries with top 10 publications were listed in Table 1. Among those 10 countries with top total publications, the USA had the largest citations (n = 10805) (Table 1), far more than other countries. In terms of citation/publication ratio, France had the top ratio despite a small number of total publications, which indicated that publications from countries were of high quality and relevance in SBA research field (Table 1). Notably, China had a large number of publications (n = 102), its citation/publication ratio (9.44) was unremarkable (Table 1), partially due to the fact that most of the publications from China were published in recent years.

Institutions with top 10 publications were listed in Table 2, which included Mayo Clinic (n = 22), University of Texas MD Cancer Center (n = 22), University of Ulsan (n = 17), Catholic University of Korea (n = 14), National Cancer Institute (n = 11), Karolinska Institution (n = 11), University of Padua (n = 10), Sungkyunkwan University (n = 10), University of Pavia (n = 10), University of Hosp (n = 10). University of Texas MD Cancer Center also had the highest citations and citation/publication ratio, which indicated that University of Texas MD Cancer Center play a central role in this field. Additionally, the collaborations between institutions are illustrated in Figure X. The Mayo Clinic, University of Texas MD Cancer Center and University of Ulsan have relatively wider collaborations than other institutions.

**Table 2:** Top 10 institutions with the most publications in research of small bowel adenocarcinoma.

Rank	Institutions	Publications	Citations	Citations/Publications
1	Mayo Clinic	22	852	38.73
2	University Texas MD Anderson Cancer Center	22	1082	49.18
3	University of Ulsan	17	315	18.53
4	Catholic University of Korea	14	276	19.71
5	National Cancer Institute (NCI)	11	254	23.09
6	Karolinska Institution	11	334	30.36
7	University of Padua	10	119	11.90
8	Sungkyunkwan University	10	239	23.90
9	University of Pavia	10	81	8.10
10	University of Hosp	10	214	21.40

Journals with top 10 publications are listed in Table 3. The top 10 most prolific journals on SBA were “World Journal Of Gastroenterology” (n = 30), “Diseases Of The Colon & Rectum” (n = 28), “International Journal of Radiation Oncology Biology Physics” (n = 18), “Journal Of Gastrointestinal Surgery” (n = 17). Among those top 10 journals, 70% of them are categorized as Q1, with *Gastrointestinal Endoscopy* Journal having the highest impact factor (IF = 10.396). Cancer had the highest citation and citation/publication ratio.

**Table 3:** Top 10 journals with the most publications in research of small bowel adenocarcinoma.

Rank	Journal	Publications	Citations	Citations/Publications	Impact factor	Quartile in category
1	World Journal of Gastroenterology	30	773	25.77	5.374	Q1
2	Diseases of the Colon & Rectum	28	684	24.43	4.412	Q1
3	International Journal of Radiation Oncology Biology Physics	18	376	20.89	8.013	Q1
4	Cancers	17	65	3.82	6.575	Q2
5	Journal of Gastrointestinal Surgery	17	358	21.06	3.267	Q1
6	American Journal of Surgical Pathology	17	764	44.94	6.298	Q1
7	Scandinavian Journal of Gastroenterology	16	327	20.44	3.027	Q2
8	Gastrointestinal Endoscopy	16	506	31.63	10.396	Q1
9	Cancer	15	1229	81.93	6.921	Q1
10	Oncology Letters	15	98	6.53	3.111	Q2

Authors with top 10 publications are listed in Table 4. The most active and productive authors with top publications included Overman Michael J (n = 17), Aparicio Thomas (n = 15), Zaanan Aziz (n = 14), Afchain Pauline (n = 9), Hong, Seung-mo (n = 9). Notably, the Overman Michael J and Aparicio Thomas also had the top 2 citations and average citations per publication ratio.

**Table 4:** Top 10 authors with the most publications in research of small bowel adenocarcinoma.

Rank	Author	Publications	Citations	Citations/Publications
1	Overman, Michaelj	17	835	49.12
2	Aparicio, Thomas	15	619	41.27
3	Zaanan, Aziz	14	457	32.64
4	Afchain, Pauline	9	348	38.67
5	Hong, Seung-Mo	9	163	18.11
6	Jun, Sun-Young	8	153	19.13
7	Pocard, Marc	8	248	31.00
8	Wolff, Roberta.	8	508	63.50
9	Ahrens, W	7	110	15.71
10	Eriksson, M	7	110	15.71

**Figure 4.**
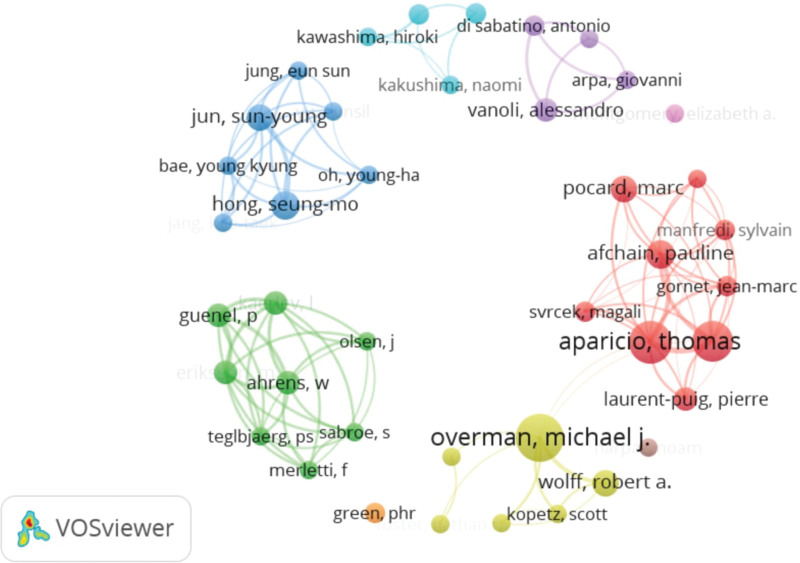
Network map of co-occurring authors in research of small bowel adenocarcinoma.

Co-authorship analysis revealed that Overman Michael J, Aparicio Thomas had a relatively wider collaboration than other authors. Interestingly, the authors tend to form a small domestic research group but they lack inter-group cooperation (Fig. 4).

The most co-cited journals were listed in Table 5, including Gastroenterology (n = 1144), followed by Cancer (n = 947), Gut (n = 805), Cancer-American Cancer Society (n = 781), Diseases of The Colon & Rectum (n = 729), Annals of Surgery (n = 714). Among the top 10 most co-cited journals, the Gut had the highest impact factor (IF = 31.793).

**Table 5:** Top 10 most co-cited journals and authors in research of small bowel adenocarcinoma.

Rank	Journal	Co-citations	Rank	Author	Co-citations
1	Gastroenterology	1144	1	OvermanMJ	418
2	American Journal of Gastroenterology	947	2	DabjaBS	209
3	Cancer	805	3	AparicioT	200
4	Gut	781	4	BilimoriaKY	177
5	Cancer-American Cancer Society	729	5	HoweJR	160
6	Diseases of The Colon & Rectum	717	6	NeugutAI	147
7	Annals of Surgery	714	7	ZaananA	122
8	Journal of Clinical Oncology	661	8	JessT	115
9	Annals of Surgical Oncology	556	9	HalfdanarsonTR	100
10	American Journal of Surgery	489	10	JemalA	94

**Figure 5.**
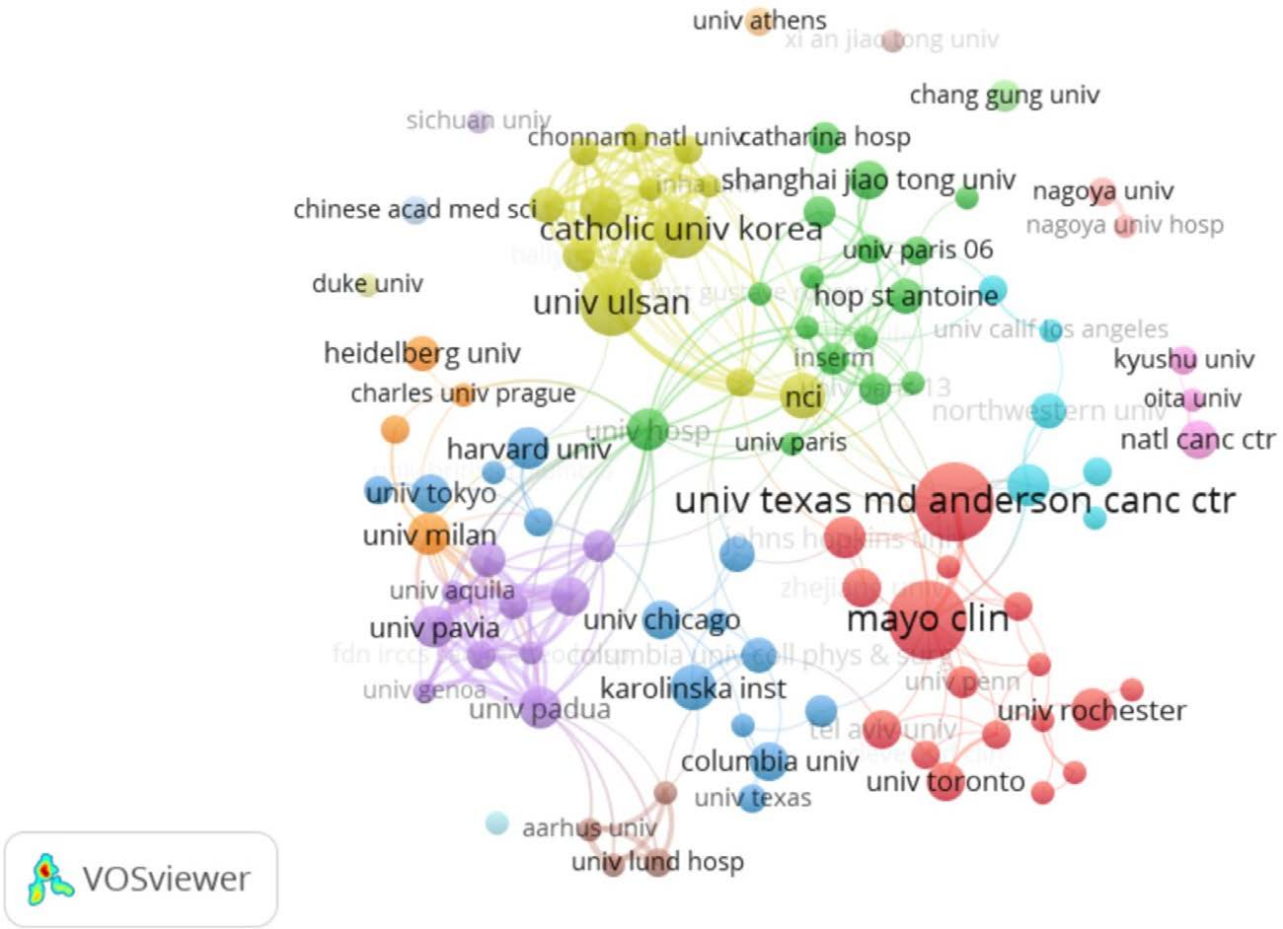
Network map of co-occurring institutions in research of small bowel adenocarcinoma.

We also performed co-citation analysis for the authors, the results are shown in Figure 5 and Table 5. We found that Overman Michael J (n = 418) was the most co-cited author, followed by Dabaja bs (n = 209), Aparicio Thomas (n = 200), Bilimoria KY (n = 177) (Table 5).

We identified the most co-cited publication through co-citations analysis of references, the results are shown in Table 6. Our results indicated that the article titled “*Adenocarcinoma of the small bowel: presentation, prognostic factors, and outcome of 217 patients*” published in Cancer by Dabaja DS in 2004 was the most co-cited publication. The second most co-cited paper was published by Bilimoria KY in 2009 in Annals of Surgery titled “*Small bowel cancer in the United States: changes in epidemiology, treatment, and survival over the last 20 years*” (Table 6).

**Table 6:** Top 5 most co-cited publications in research of small bowel adenocarcinoma.

Rank	Reference
1	Dabaja, Bouthaina S et al “Adenocarcinoma of the small bowel: presentation, prognostic factors, and outcome of 217 patients.” Cancer vol. 101,3 (2004): 518–26. doi:10.1002/cncr.20404
2	Bilimoria, Karl Y et al “Small bowel cancer in the United States: changes in epidemiology, treatment, and survival over the last 20 years.” Annals of Surgery vol. 249,1 (2009): 63–71. doi:10.1097/SLA.0b013e31818e4641
3	Howe, J R et al “The American College of Surgeons Commission on Cancer and the American Cancer Society. Adenocarcinoma of the small bowel: review of the National Cancer Data Base, 1985–1995.” Cancer vol. 86,12 (1999): 2693–706. doi:10.1002/(sici)1097–0142(19991215)86:12 < 2693::aid-cncr14 > 3.0.co;2-u
4	Overman, Michael J et al “Phase II study of capecitabine and oxaliplatin for advanced adenocarcinoma of the small bowel and ampulla of Vater.” Journal of Clinical Oncology: official journal of the American Society of Clinical Oncology vol. 27,16 (2009): 2598–603. doi:10.1200/JCO.2008.19.7145
5	Halfdanarson, Thorvardur R et al “A single-institution experience with 491 cases of small bowel adenocarcinoma.” American Journal of Surgery vol. 199,6 (2010): 797–803. doi:10.1016/j.amjsurg.2009.05.037

**Figure 6.**
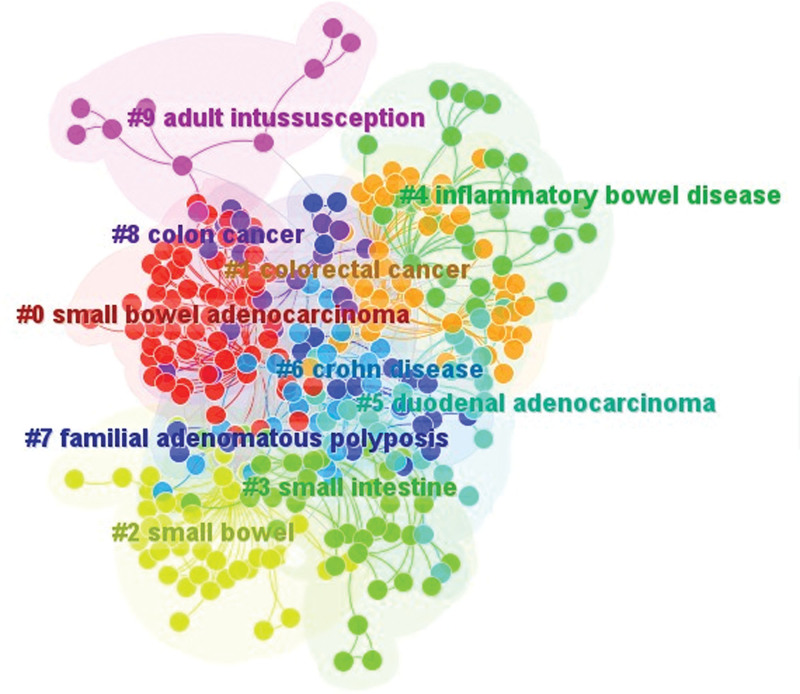
Visualization of keywords clustering network based on Citespace.

Keyword co-occurrence analysis could identify research topics that have gained attention in a given period. Our results showed that SBA was the most frequent keyword (n = 117) in our analysis period, followed by small bowel (n = 74), Crohn disease (n = 72), small intestine (n = 68), inflammatory bowel disease (n = 39), colorectal cancer (n = 32), small bowel cancer (n = 30) (Table 7). The keyword with top centrality was SBA (centrality = 0.27) (Table 7). We also performed keyword clustering analysis, results of which indicated those keywords could be generally divided into 10 clusters, namely “small bowel adenocarcinoma,” “colorectal cancer,” “small bowel,” “small intestine,” “inflammatory bowel disease,” “duodenum adenocarcinoma,” “Crohn disease,” “familial adenomatous polyposis,” “colon cancer,” “adult intussusception” (Fig. 6). Notably, 2 cluster of keyword that were associated to inflammatory bowel disease appeared in our result, which indicated that inflammatory bowel disease was frequently mentioned or discussed in SBA research (Fig. 6).

**Table 7:** Top 10 keywords with most frequency in research of small bowel adenocarcinoma

Rank	Keyword	Counts	Centrality
1	Small bowel adenocarcinoma	177	0.27
2	Small bowl	74	0.13
3	Crohn disease	72	0.11
4	Small intestine	68	0.09
5	Inflammatory bowel disease	39	0.10
6	Colorectal cancer	31	0.10
7	Small bowel cancer	30	0.10
8	Celiac disease	26	0.04
9	Capsule endoscopy	22	0.05
10	Colon cancer	22	0.08

**Figure 7.**
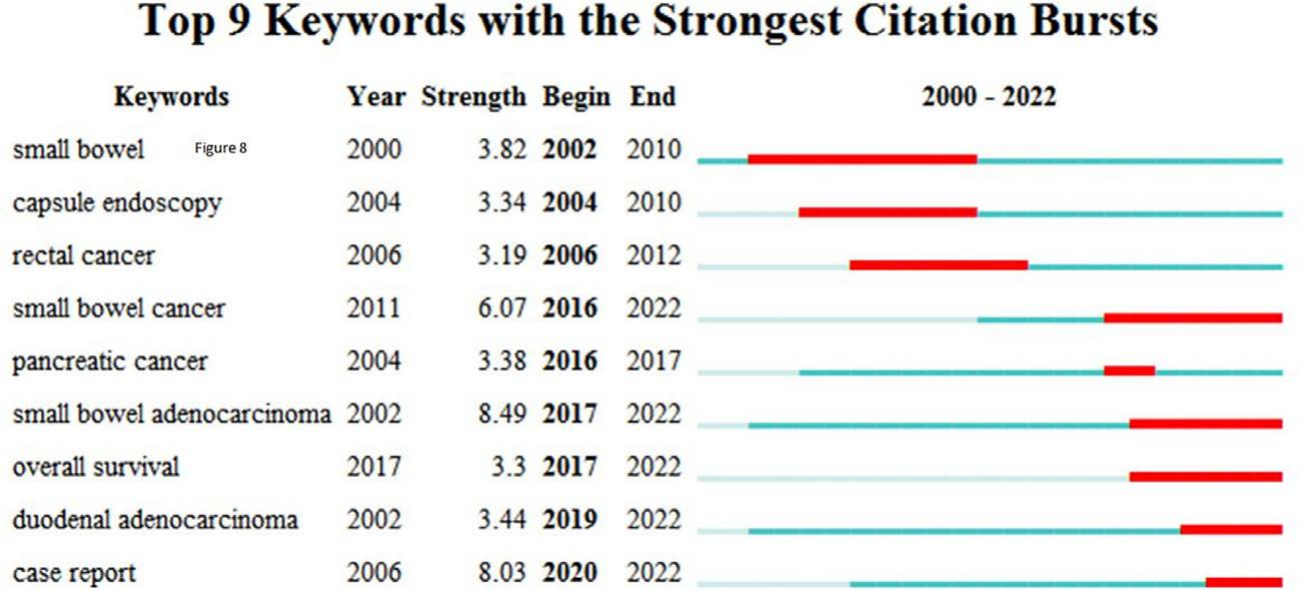
Top 9 keywords with strongest citation bursts in research of small bowel adenocarcinoma from 2000 to 2022.

Keyword citation burst analysis could reveal hot topics in this field over time. Our results indicated that approximately 2 periods could be identified over the last 2 decades. In the first period from 2002 to 2012, researchers mainly focused on small bowel, capsule endoscopy, rectal cancer (Fig. 7). While in the second period from 2016 to 2022, researchers started to focus on small bowel cancer, pancreatic cancer, SBA, overall survival, duodenal adenocarcinoma and case report (Fig. 7). Notably, case report had citation burst from 2020 to 2022, which indicated that many individual cases of clinical significance were emerging in recent years (Fig. 7).

## 4. Discussion

Over the past 2 decades, significant research has been conducted on SBA, a rare type of cancer that arises in the small intestine. In this bibliometric analysis, we aim to provide an overview of the landscape of SBA research, focusing on trends and knowledge gaps related to time, geography, and research topics.

Our analysis reveals an increased trend of annual publications on SBA, although this trend is still unremarkable. One of the primary reasons for this is the low incidence of SBA, which makes it challenging to conduct large cohort studies. Nevertheless, the research on SBA has been steadily increasing and has led to insights into the diagnosis, treatment and management of SBA.

We found that France and the USA are the leading countries in the field of SBA research, contributing the largest proportion of publications in this area. Additionally, these countries collaborate extensively with other nations to advance the research. Notably, France and the USA have published their guidelines,^[14,15]^ which is a significant milestone in the management of SBA patients. However, there are still many clinical questions that remain unresolved, and no clear recommendations have been presented as of yet. Thus, there is a need for further research and evidence-based guidelines in this field.

Co-authorship analysis revealed 2 research groups in France and the USA that have contributed significantly to the SBA research publications. Within the top yield authors and institutions, most of them are from these 2 countries. This finding could be attributed to the high level of interdisciplinary collaboration, strong funding of research, and advanced technology available in these countries.

The most co-cited article was published by Bouthaina S Dabaja in *Cancer* in 2004.^[16]^ in this article, the authors retrospectively reviewed records of 217 patients with SBA and focused on the presentation, prognostic factors, treatment modalities, and outcomes of those patients. They found that the most frequent origin was duodenum (52%), followed by jejunum (25%) and ileum (13%). Similarly, the most frequently performed operation was Whipple procedure (17%). Discouragingly, they found the median overall survival time was only 20 months and the 5-year overall survival rate was 26%. This paper published in early 21^st^ century provided a general profile of small adenocarcinoma and set the tone for the following studies. The second most co-cited article was published by Karl Y Bilimoria in 2009 in *Annals of Surgery.*^[17]^ The authors reviewed the data of 843 patients with small bowel malignancies from the National Cancer Data Base (NCDB, 1985–2005) and the Surveillance Epidemiology and End Results (SEER, 1973–2004) database. They found SBA accounted for 36.9%. Interestingly, their analysis showed that adjuvant chemotherapy for adenocarcinoma increased from 8.1% in 1985 to 23.8% in 2005 but the 5-year survival after resection remained unchanged over time. Based on this finding, the authors suggested that efforts and emphasis should be put on novel therapeutic options development and investigation.

The keywords analysis revealed the trend of research hotspots over the last 2 decades. Top 10 most frequent keywords identified in our analysis included colorectal cancer as well as inflammatory bowel disease, which indicated that those 2 keywords were frequently mentioned in SBA study and were closely associated with SBA. For instance, a study has shown that the MSS SBA more similar to colorectal than to gastric cancer, based on the 27 genome-wide DNA copy number profiles.^[18]^ Moreover, a recent systemic meta-analysis indicated that SBA with Crohn disease had poor 5-year overall survival.^[19]^

Recently, our analysis indicated that there are citation bursts of case reports of SBA from 2020 to 2022, which suggests that many intriguing and informing clinical cases were published and discussed by doctors. This also indicated that individualized diagnosis and management of SBA are under investigation.

Admittedly, there are some limitations to this study. Firstly, this only included English publications from Web of Science Core Collection, which inevitably introduced selection bias, given that a large number of studies were published in other languages. Secondly, this study only focused on citation metrics and is incapable of highlighting recent breathtaking publications just because the citation metrics are time-dependent, which indicates that recent publications are less likely to be cited.

## 5. Conclusion

This bibliometric analysis provides a comprehensive overview of the trends and gaps in the research landscape of SBA. Despite the challenges faced, researchers from USA, Japan and China have made significant contributions to the field of SBA research. Further research is on urgent need to clarify unresolved clinical questions, to develop evidence-based guidelines, and advance the understanding and treatment of SBA.

## Authors contributions

**Conceptualization:** Guang Fu.

**Data curation:** Li Li, Shao Zhang.

**Formal analysis:** Li Li, Shao Zhang, Guang Fu.

**Writing – original draft:** Li Li, Guang Fu.

**Writing – review & editing:** Guang Fu.
